# Letter to the Editor: Expected Improvement in Structure–Function Agreement With Macular Displacement Models

**DOI:** 10.1167/tvst.11.10.14

**Published:** 2022-10-11

**Authors:** Giovanni Montesano, David F. Garway-Heath, David P. Crabb

**Affiliations:** 1 Department of Optometry and Visual Sciences, City University of London, London, UK; 2 NIHR Biomedical Research Centre, Moorfields Eye Hospital NHS Foundation Trust and UCL Institute of Ophthalmology, London, UK. e-mail: giovmontesano@gmail.com

##  

We read with interest the paper by Tong et al.^1^ recently published in *Translational Vision Science & Technology*. They analyzed the effect of considering retinal ganglion cell (RGC) displacement on structure–function agreement in the macula in patients with glaucoma. For their main analysis, they assessed the agreement between 10-2 visual field (VF) defects on pattern deviation maps and structural defects, identified with macular optical coherence tomography (OCT) volumes using probability deviation maps of the ganglion cell layer thickness. For VF locations, they used the average displacement calculated by Drasdo et al.^2^^,^^3^ and compared the structure–function agreement with and without displacement. Interestingly, but perhaps not surprisingly, they found no improvement in the agreement when accounting for displacement. This is in keeping with previous results.^4^ The authors offer various explanations for this result and importantly note that “glaucomatous structural damage at the macula typically manifests as broad arcuate defects, even in early stages of disease. If defects are wider than the magnitude of displacement, then similar OCT measurements will be observed regardless of whether data are extracted with displacement applied or not.” This is an interesting proposition that warrants additional considerations.

**Figure 1.**
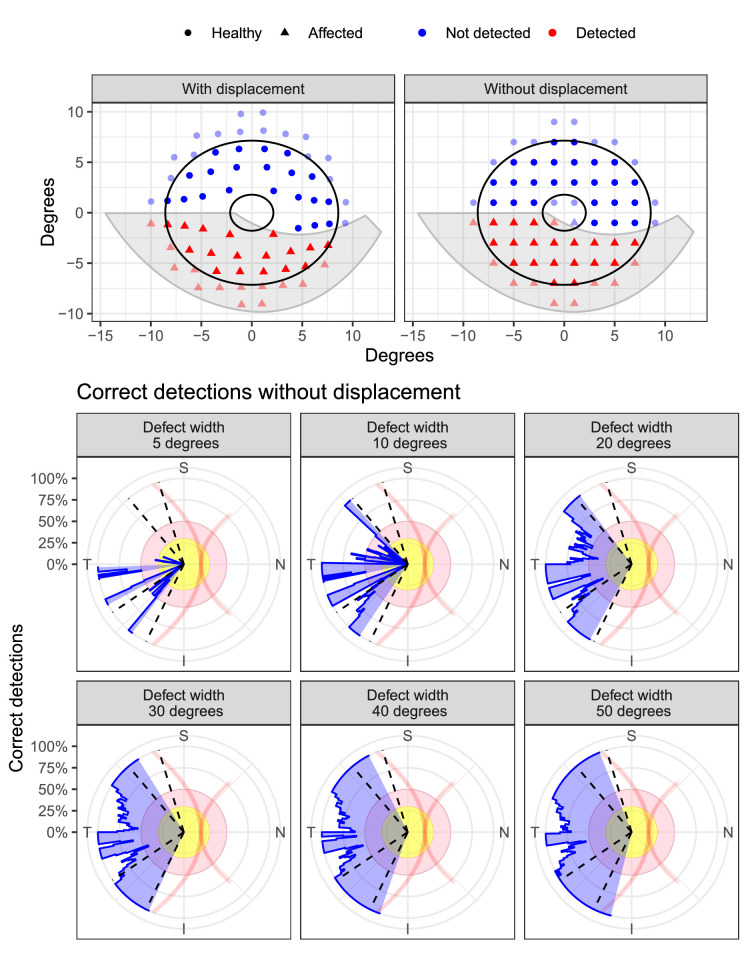
The *top panel* shows an example of the calculation for a defect with 30° angular width. Correct detections are identified as *red triangles*, meaning that the location is both mapped to a structural defect and actually affected. Only locations within the annulus delimited by the *black ellipses* are considered. The *bottom panels* represent the percentage of correct detections (*blue-shaded bar chart*), without accounting for displacement, at different positions around the optic nerve head and different angular widths of the defect. The maximum extent of the polar axes corresponds 100% (perfect detection, no different from using the correct displacement model). The *dashed lines* delimit the sectors where macular bundle defects are more commonly found. I, inferior; T, temporal; S, superior; N, nasal. From Leung et al.^9^

We ran some simple theoretical calculations to determine the expected improvement in measured structure–function agreement when accounting for RGC displacement, assuming perfect structure–function correlation and absolute accuracy of the Drasdo model. We used implementations of the Drasdo model^2^ and Jansonius’ model of retinal nerve fiber (RNF) trajectories^5^ freely available with the visualFields^6^ package for R. Using Jansonius’ model, we generated RNF bundle defects of varying angular widths at different angles around the optic nerve head. Locations in the 10-2 grid were considered as being actually “affected” if their position, after being displaced with the Drasdo model, was included in the defect. This meant that accounting for displacement always resulted in perfect structure–function agreement. We then identified the locations that were detected as being contained within the defect when no displacement was applied and compared the results. We limited our calculations to the same annular region used by Tong et al.^1^ From the example in Figure 1, which shows a defect approximately corresponding to the macular vulnerability zone,^7^ it is immediately evident that no improvement in agreement would be observed with displacement, as the only location misclassified without displacement would be located within the parafoveal region and therefore excluded from the calculation. This is more systematically shown in the bottom panels in Figure 1. Not accounting for displacement leads to correct detection of affected locations for most angular positions of the RNF defect when the defect is 30° or more in angular width, with perfect detection in the superior–temporal and inferior–temporal sectors, which are the most commonly affected.^7^^,^^8^ This has been recently confirmed by Leung et al.,^9^ who looked at the distribution of macular bundle defects in glaucoma. Meaningful discrepancies between displaced and not displaced stimuli are only found for extremely localized defects (20° or less), which are, however, much rarer^7^^,^^8^ and more difficult to quantify.

This modeling exercise helps illustrate the theoretical expectation when testing the accuracy of displacement models. In fact, the chance of increasing accuracy by accounting for displacement is extremely slim for most commonly occurring macular defects, even when the exact displacement is known and perfect structure–function agreement can be assumed. In reality, other factors can further decrease the chances of detecting a difference, such as test–retest variability in structural and functional tests, imperfect structure–function agreement, imprecise structure–function mapping due to eye movements and eccentric fixation, and, importantly, inaccuracy of the displacement model itself from errors in histology measurements of the length of the Henle fibers and RGC density in flat-mounted retinas.^4^ Moreover, the limited area analyzed with the structural probability maps means that, on the one hand, more locations are excluded when displacement is considered, reducing the precision of the estimates; on the other hand, the area with the greatest displacement (parafovea) is excluded when displacement is not considered, biasing the results.

We would also like to point out some methodological aspects of the work by Tong et al.^1^ that could have had an effect on the results, albeit likely very small:

(1) The displacement model proposed by Drasdo et al.^2^^,^^3^ is not radially symmetric, unlike that used by the authors, and implementations of the correct model are now freely available.^3^^,^^6^
(2) Axial length and ocular magnification can have an effect on the amount of displacement, especially in highly myopic eyes.
(3) The area covered by each stimulus used to measure local RGC layer thickness (secondary analysis in Tong et al.^1^) should not be calculated by simply displacing its center; instead, each point of the perimeter of the stimulus should be distorted with the model, leading to ovoidal shapes. We have previously shown this to be the only method that provides correct estimates of local RGC counts.^3^ The effect would be smaller when considering local average thickness, but this is yet another potential source of imprecision.

**Figure 2.**
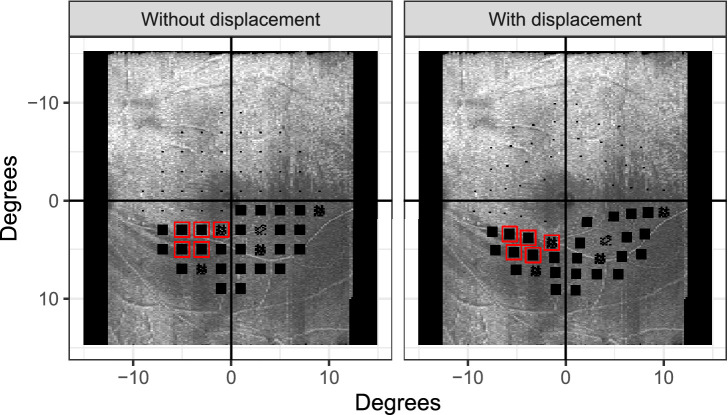
Pattern deviation probability map of a 10-2 full-threshold test mapped to an en face reflectivity map of the RNF layer, with and without displacement. The locations highlighted in *red* are those where the structure–function agreement was improved by displacement.

However, most of these aspects, especially points 1 and 3, have been addressed and corrected by the same group in a very recent publication.^10^ All that said, we believe that the results in Tong et al.^1^ generally hold true and are mainly a consequence of the prevalent size and topography of macular glaucomatous defects. The results should not, however, lead to dismissing the existence and importance of RGC displacement, which is simply a well-established fact of nature. Tong et al.^1^ also give a similar interpretation for their findings. Perhaps more effort should be devoted to testing these models in eyes where the effect of displacement can be more clearly observed or by arranging the perimetric tests to specifically probe the edges of RNF defects. En face imaging allows accurate qualitative evaluation of RNF defect topography and could be used for this scope. The example in Figure 2 shows an application of this principle in an eye in which applying Drasdo's displacement causes some diseased locations to be moved from a healthy RNF layer bundle to a bundle defect, improving structure–function agreement. Enriching test samples with such cases or customizing perimetric test locations would allow a more systematic investigation of this important research question.
